# Evaluation of dietary crude protein and non-starch polysaccharidase on nursery pig performance, nitrogen retention, hindgut fermentation and health

**DOI:** 10.1093/tas/txag090

**Published:** 2026-07-02

**Authors:** Mitchell J Nisley, Brian J Kerr, Sarah C Pearce, Chris Sparks, Nicholas K Gabler

**Affiliations:** Department of Animal Science, Iowa State University, Ames, IA 50011, United States; USDA-ARS National Laboratory for Agriculture and the Environment, Ames, IA 50011, United States; USDA-ARS National Laboratory for Agriculture and the Environment, Ames, IA 50011, United States; Department of Animal Science, Iowa State University, Ames, IA 50011, United States; Department of Animal Science, Iowa State University, Ames, IA 50011, United States

**Keywords:** nitrogen, non-starch polysaccharidase, nursery, nutrition, pigs, protein

## Abstract

Reducing dietary crude protein (CP) in nursery pig diets is a strategy to decrease post-weaning diarrhea (PWD), optimize feed costs, and reduce nitrogen excretion. One way by which high CP (HCP) diets increase PWD involve potentially cytotoxic proteolytic fermentation metabolites produced in the hindgut from unabsorbed proteins. Increasing dietary fiber fermentability with carbohydrase inclusion may mitigate this modality by shifting microbial fermentation towards carbohydrate substrate. To evaluate these interactions, a 3 × 2 factorial design assessed pig performance, nitrogen retention, hindgut fermentation, and health outcomes using high- and low- CP diets with or without a non-starch polysaccharidase (NSPase; xylanase). A total of 792 newly weaned 17 to 22- d-old mixed sex pigs (6.2 ± 0.12 kg body weight [BW]) were randomly allotted to three CP diets with or without NSPase (*n* = 12 pens/treatment), fed in three phases (10, 11, and 21 d). The CP and Lys per diet and phase were: (1) 16% CP containing 1.20, 1.15, and 1.10% standardized ileal digestible (SID) Lys per phase (LCP1.2); (2) 17% CP containing 1.40, 1.35, and 1.30% SID Lys (LCP1.4); (3) 24% CP containing 1.40, 1.35, and 1.30% SID Lys (HCP1.4). On d 10, one barrow per pen (*n* = 12 pigs/treatment) was moved to metabolism crates and fed at ∼5% of their average BW for 7 d on phase 2 diets, after which total fecal, and urine output were collected over a 4-d period for nitrogen retention analysis. On d 21, pigs utilized for metabolism evaluation were euthanized to collect intestinal tissue and luminal contents. The highest overall average daily gain and feed efficiency were observed in pigs fed HCP1.4 (both *P* < 0.0001). During phase 1, a tendency for a diet × NSPase interaction on PWD incidence was observed (*P* = 0.063), as NSPase numerically decreased incidence in HCP1.4 pigs but increased it in LCP1.4 and LCP1.2 pigs; overall, HCP1.4 pigs had greater PWD incidence than LCP1.4 and LCP1.2 pigs (*P* < 0.045). Pigs fed HCP1.4 diets observed increased nitrogen excretion and retention (g/d) when compared pigs fed either LCP diet (both *P* < 0.001, respectively). Pigs fed HCP1.4 had the highest production of total volatile fatty acids (VFA, *P* = 0.027). Polyamines cadaverine and putrescine were unaffected by CP (*P* > 0.05), but NSPase tended to reduce cadaverine production in all pigs (*P* = 0.079). Herein, pigs fed HCP diets observed improved performance concurrently with increased nitrogen excretion and PWD incidence.

## Teaser

Dietary crude protein levels influence nursery pig growth, nitrogen retention, and hindgut fermentation, while non-starch polysaccharidases may help shift fermentation dynamics and reduce post-weaning diarrhea associated with high protein intake.

## Introduction

The inclusion rate of crude protein (CP) in early nursery diets of pigs has been subject to scrutiny due to the complex nature of the weaning transition and the potential for enteric disturbances. Typically, “high CP” (HCP) diets in nursery diets would constitute 21% to 24% CP and “low CP” (LCP) diets would be considered 16% to 18% CP (Rocha et al. 2022). To achieve the latter, crystalline amino acids (AA) and specialty proteins or reduced SID Lys curves (AA requirements relative to energy [NRC 2012]) are often utilized by nutritionists to reduce CP content of early nursery diets. The rationale behind these diet formulation changes is driven by evidence linking reduced CP to improved enteric health (Pieper et al. 2016) and decreased environmental nitrogen excretion (Kerr and Easter 1995; Portejoie et al. 2004; Shurson and Kerr 2023). Moreover, HCP diets have consistently elicited more frequent and prolonged post-weaning diarrhea (PWD) occurrences in young pigs when compared to lower CP diets (Kim et al. 2023; Limbach et al. 2021; Lynegaard et al. 2021). One proposed mechanism by which elevated dietary CP intake contributes to PWD in young pigs is through increased hindgut fermentation of unabsorbed protein (Zhang et al. 2020).

Given that excessive dietary protein potentiates proteolytic fermentation and PWD, strategies that shift hindgut fermentation toward saccharolytic pathways are of increasing interest. One such approach involves the inclusion of non-starch polysaccharidases (NSPase), which enhance fiber degradation and nutrient utilization (Chen et al. 2020; Liu et al. 2019). Xylanases are a type of NSPase that targets β-1,4-glycosidic bonds between xylose residues withing xylan backbones, releasing smaller polysaccharides, pentoses, and oligosaccharides available for utilization by the pig and resident bacteria (Petry and Patience 2020). Improving non-starch polysaccharide (NSP) digestion in the foregut through NSPase supplementation may increase the availability of fermentable carbohydrates in the hindgut, where colonic microbiota are known to favor saccharolytic over proteolytic fermentation pathways (Macfarlane et al. 1992), consequently reducing colonic proteolytic fermentation and attenuating PWD.

Therefore, the objective of this study was to evaluate the effects of industry-relevant high- and low- CP diets, with or without NSPase supplementation, on nursery pig performance, nitrogen balance, hindgut fermentation, and markers of intestinal health. It was hypothesized that NSPase supplementation would improve feed efficiency, alter hindgut fermentation, and reduce PWD incidence regardless of dietary CP level. Next, it was hypothesized that pigs fed HCP diets would incur greater incidences of PWD, proteolytic fermentation, and nitrogen excretion relative to pigs fed LCP diets, while maintaining similar performance. Finally, it was hypothesized that pigs fed LCP diets formulated with a reduced SID AA: energy ratio would have attenuated performance compared to those fed LCP diets meeting NRC (2012) SID AA: energy recommendations.

## Materials and methods

All animal procedures were approved by the Iowa State University Institutional Animal Care and Use Committee (IACUC protocol #21–125).

### Animal, housing, and dietary treatments

Pigs were sourced from a commercial sow farm in which they were weaned at 19–21 d of age and transported ∼5 h to the Iowa State University Swine Nutrition Research Farm (Ames, IA). Over two replicates, a total of 792 mixed sex pigs [6.2 ± 0.12 kg body weight (BW), Camborough 1050 × 337, (PIC, Hendersonville, TN)] were randomly allotted to a 3 × 2 factorial arrangement that consisted of three diets with or without a NSPase (12 pens/treatment, 11 pigs/pen, 6 barrows and 5 gilts per pen). All diets were fed in three phases consisting of 10 d, 11 d, and 21 d over a 42-d nursery period. The CP and SID Lys per diet and phase were: (1) 16% CP containing [1.20, 1.15, and 1.10% SID Lys per phase (LCP1.2)]; (2) 17% CP containing [1.40, 1.35, and 1.30% SID Lys (LCP1.4)]; and (3) 24% CP containing [1.40, 1.35, and 1.30% SID Lys (HCP1.4)]. To separate the effects of CP level from those of SID Lys, only the HCP1.4 and LCP1.4 diets were standardized to a Lys: energy ratio. All diets were standardized to an SID AA: Lys ratio, enabling LCP1.2 to maintain balanced AA ratios while remaining below total NRC (2012) AA recommendations. The NSPase utilized was included at 0.05% (3000 EPU/g) and consisted of a fiber degrading enzymatic complex based on β-1,4 xylanase derived from a *Trichoderma citrinoviride* strain. Diets were isocaloric corn-soybean meal based (Tables 1–3 for Phase 1, 2, and 3, respectively). All diets were provided ad libitum throughout the 42-d trial and water was offered via two water nipples per pen.

**Table 1:** Phase 1 diet composition, as-fed.

Ingredient, %	LCP1.2	LCP1.4	HCP1.4
**Corn, ground**	64.31	63.88	41.49
**Soybean meal, 46.5% crude protein**	11.85	10.00	37.45
**Plasma, spray-dried**	2.50	2.50	2.50
**Fish meal, menhaden**	13.89	2.50	–
**Whey, dried**	2.50	13.89	13.89
**Casein**	–	2.00	–
**Soybean oil**	1.25	1.00	1.81
**Limestone**	0.67	0.68	0.87
**Monocalcium phosphate, 21%**	0.67	0.65	0.52
**Salt**	0.39	0.39	0.41
**L-Lys HCl**	0.48	0.63	0.08
**DL-Met**	0.14	0.21	0.08
**L-Thr**	0.14	0.21	–
**L-Trp**	0.06	0.08	–
**L-Val**	0.12	0.28	–
**L-Ile**	0.13	0.20	–
**Phytase^a^**	0.02	0.02	0.02
**Vitamin premix^b^**	0.25	0.25	0.25
**Trace mineral premix^c^**	0.15	0.15	0.15
**Zinc oxide, 72% Zn**	0.40	0.40	0.40
**Copper sulfate, 25.2% Cu**	0.08	0.08	0.08
**NSPase^d^**	0.05*	0.05*	0.05*
***Calculated nutrient composition***			
**Crude protein, %**	16.00	17.30	24.00
**ME^e^, kcal/kg**	3396	3396	3396
**SID^f^ Lys, %**	1.20	1.40	1.40
**SID Lys: ME, g/Mcal**	3.53	4.12	4.12
**SID Lys: CP**	7.50	8.09	5.83
***SID amino acid: Lys***			
**Thr**	0.60	0.60	0.62
**Met**	0.33	0.36	0.30
**Met + Cys**	0.55	0.55	0.56
**Trp**	0.19	0.19	0.21
**Ile**	0.59	0.59	0.67
**Val**	0.67	0.66	0.74
**Phosphorus, available %**	0.55	0.56	0.48
**Calcium, total %**	0.65	0.65	0.65
**Acid detergent fiber, %**	2.30	2.17	3.39
**Neutral detergent fiber, %**	5.62	5.49	5.20

a Optiphos 2500; Huvepharma, Peachtree City, GA, USA. Minimum activity of 250 phytase units (FTU)/kg complete feed.

b Vitamin premix provided 6,125 IU vitamin A, 700 IU vitamin D_3_, 50 IU vitamin E, 30 mg vitamin K, 0.05 mg vitamin B_12_, 11 mg riboflavin, 56 mg niacin, and 27 mg pantothenic acid per kilogram of diet.

c Trace mineral premix provided 9 mg Cu (as CuSO_4_), 120 mg Fe (as FeSO_4_), 0.21 mg I (as Ca(IO_3_)_2_), 6.75 mg Mn (as MnSO_4_), 120 mg Zn (as ZnSO_4_), and 0.23 mg Se (as Na_2_SeO_3_) per kilogram of diet.

d Xylanase (500 g/t; 1500 EPU/kg of whole diet).

e ME, Metabolizable energy.

f SID, Standardized ileal digestibility.

* Provided with or without basal diet.

**Table 2:** Phase 2 diet composition, as-fed.

Ingredient, %	LCP1.2 (*1.15*)	LCP1.4 (*1.35*)	HCP1.4 (*1.35*)
**Corn, ground**	72.86	70.06	51.28
**Soybean meal, 46.5% crude protein**	18.00	15.00	38.00
**Plasma, spray-dried**	–	1.50	1.50
**Fish meal, menhaden**	2.48	4.52	–
**Whey, dried**	–	3.47	3.47
**Soybean oil**	2.13	1.31	2.30
**Limestone**	0.70	0.56	0.90
**Monocalcium phosphate, 21%**	1.00	0.62	0.81
**Salt**	0.89	0.68	0.73
**L-Lys HCl**	0.54	0.63	0.17
**DL-Met**	0.14	0.20	0.07
**L-Thr**	0.18	0.22	0.01
**L-Trp**	0.07	0.09	–
**L-Val**	0.13	0.19	–
**L-Ile**	0.12	0.19	–
**Phytase^a^**	0.02	0.02	0.02
**Vitamin premix^b^**	0.25	0.25	0.25
**Trace mineral premix^c^**	0.15	0.15	0.15
**Zinc oxide, 72% Zn**	0.26	0.26	0.26
**Copper sulfate, 25.2% Cu**	0.08	0.08	0.08
**NSPase^d^**	0.05*	0.05*	0.05*
***Calculated nutrient composition***			
**Crude protein, %**	16.00	17.50	23.70
**ME^e^, kcal/kg**	3,396	3,396	3,396
**SID^f^ Lys, %**	1.15	1.35	1.35
**SID Lys: ME, g/Mcal**	3.39	3.98	3.97
**SID Lys: CP**	7.19	7.71	5.70
***SID amino acid: Lys***			
**Thr**	0.60	0.60	0.60
**Met**	0.35	0.36	0.29
**Met + Cys**	0.55	0.55	0.56
**Trp**	0.19	0.19	0.20
**Ile**	0.59	0.59	0.65
**Val**	0.67	0.67	0.73
**Phosphorus, available, %**	0.49	0.65	0.45
**Calcium, total %**	0.65	0.51	0.65
**Acid detergent fiber, %**	2.89	2.63	2.66
**Neutral detergent fiber, %**	6.61	6.23	6.00
***Analyzed nutrient composition***			
**Crude protein (Rep. 1), %**	15.7	17.2	23.0
**Crude protein (Rep. 2), %**	16.6	18.2	23.6

a Optiphos 2500; Huvepharma, Peachtree City, GA, USA. Minimum activity of 250 phytase units (FTU)/kg complete feed.

b Vitamin premix provided 6,125 IU vitamin A, 700 IU vitamin D_3_, 50 IU vitamin E, 30 mg vitamin K, 0.05 mg vitamin B_12_, 11 mg riboflavin, 56 mg niacin, and 27 mg pantothenic acid per kilogram of diet.

c Trace mineral premix provided 9 mg Cu (as CuSO_4_), 120 mg Fe (as FeSO_4_), 0.21 mg I (as Ca(IO_3_)_2_), 6.75 mg Mn (as MnSO_4_), 120 mg Zn (as ZnSO_4_), and 0.23 mg Se (as Na_2_SeO_3_) per kilogram of diet.

d Xylanase (500 g/t; 1,500 EPU/kg of whole diet).

e ME, Metabolizable energy.

f SID, Standardized ileal digestibility.

* Provided with or without basal diet.

**Table 3:** Phase 3 diet composition, as-fed.

Ingredient, %	LCP1.2 (*1.1*)	LCP1.4 (*1.3*)	HCP1.4 (*1.3*)
**Corn, ground**	71.22	76.06	51.57
**Soybean meal, 46.5% crude protein**	22.00	15.00	43.62
**Fish meal, menhaden**	–	3.49	–
**Soybean oil**	2.31	1.21	2.14
**Limestone**	0.89	0.63	0.91
**Monocalcium phosphate, 21%**	1.25	0.91	0.83
**Salt**	0.91	0.40	0.40
**L-Lys HCl**	0.49	0.77	0.07
**DL-Met**	0.13	0.23	0.04
**L-Thr**	0.15	0.29	–
**L-Trp**	0.05	0.11	–
**L-Ile**	0.10	0.23	–
**L-Val**	0.08	0.25	–
**Phytase^a^**	0.02	0.02	0.02
**Vitamin premix^b^**	0.25	0.25	0.25
**Trace mineral premix^c^**	0.15	0.15	0.15
**NSPase^d^**	0.05*	0.05*	0.05*
***Calculated nutrient composition***			
**Crude protein, %**	16.05	16.00	24.00
**ME^e^, kcal/kg**	3,396	3,396	3,396
**SID^f^ Lys, %**	1.10	1.30	1.30
**SID Lys: ME, g/Mcal**	3.24	3.83	3.83
**SID Lys: CP**	6.85	8.13	5.42
***SID amino acid: Lys***			
**Thr**	0.60	0.60	0.63
**Met**	0.33	0.38	0.29
**Met + Cys**	0.55	0.55	0.55
**Trp**	0.19	0.19	0.21
**Ile**	0.59	0.59	0.72
**Val**	0.67	0.67	0.77
**Phosphorus, available, %**	0.48	0.49	0.41
**Calcium, total %**	0.65	0.65	0.65
**Acid detergent fiber, %**	3.11	2.78	4.02
**Neutral detergent fiber, %**	6.69	6.70	6.31

a Optiphos 2500; Huvepharma, Peachtree City, GA, USA. Minimum activity of 250 phytase units (FTU)/kg complete feed.

b Vitamin premix provided 6125 IU vitamin A, 700 IU vitamin D_3_, 50 IU vitamin E, 30 mg vitamin K, 0.05 mg vitamin B_12_, 11 mg riboflavin, 56 mg niacin, and 27 mg pantothenic acid per kilogram of diet.

c Trace mineral premix provided 9 mg Cu (as CuSO_4_), 120 mg Fe (as FeSO_4_), 0.21 mg I (as Ca(IO_3_)_2_), 6.75 mg Mn (as MnSO_4_), 120 mg Zn (as ZnSO_4_), and 0.23 mg Se (as Na_2_SeO_3_) per kilogram of diet.

d Xylanase (500 g/t; 1,500 EPU/kg of whole diet).

e ME, Metabolizable energy.

f SID, Standardized ileal digestibility.

* Provided with or without basal diet.

Individual pig BW and whole pen feed disappearance were recorded on phase changes (d 0, 10, 21, and 42) to calculate pen average daily gain (ADG), average daily feed intake (ADFI), and feed efficiency (G:F). Pen fecal consistency scores were evaluated daily from d 0–21 and 3 × wk for the final 21 d. Fecal consistency was assessed as previously described (Nisley et al. 2025). Population-pooled fecal samples were obtained on d 4 and sent to the Iowa State University Veterinary Diagnostic Laboratory for routine pathogen screening. Removals and mortalities were recorded, with pigs failing to thrive removed by farm staff to minimize investigator bias.

### Digestibility and nitrogen retention evaluation

On d 10, at the first dietary phase change, one barrow per pen (average pen BW) was selected and moved to metabolism crates and fed 5% of their average BW of their respective phase 2 diet (Table 2) for 7 d, allowing for diet and environment acclimation. The stainless-steel metabolism crates measured 0.53 m × 0.71 m. Meals were provided twice daily at 0800 and 2000 h. Water was provided ad libitum via nipple cup. A 12-h light cycle was provided and ambient temperature in the room was maintained at thermoneutral temperatures (25–31°C). After 7 d of acclimation, total fecal and urine collection was undertaken for 4 d. Urine collection buckets were spiked with 8 mL of 6 N hydrochloric acid as a stabilizer for ammonia volatilization and to prevent microbe proliferation. Urine and total fecal output were collected twice daily and stored at −8°C until processing. At the end of the collection period, total urine samples were weighed, homogenized, and subsampled for future analysis. Total fecal collections were dried at 75°C, weighed, then ground through a 2-mm screen before being homogenized, subsampled, and stored in desiccators. Pigs were weighed after the collection period and total feed refusals were recorded over the 4-d period.

Gross energy (GE) of feed and feces were analyzed utilizing an isoperibol bomb calorimeter (Model 6200, Parr Instrument Company, Moline, IL) with benzoic acid utilized as a standard. For feed and fecal samples, 1.0 g of sample was pelleted prior to determination of GE. The GE of the diet was then multiplied by the total feed intake over the 4-d collection period and divided by 4 to determine daily GE intake. Apparent digestibility coefficients of GE were calculated by subtracting the GE of feces from the GE of intake and dividing by the GE intake. Nitrogen content of feed, feces, and urine was determined using a thermo-combustion analyzer (Variomax; Elementar Analysensysteme GmbH, Hanau, Germany). Apparent total tract digestibility (ATTD) coefficients of nitrogen were calculated as previously described (Adeola 2001). The nitrogen intake, nitrogen retained, and nitrogen retention coefficients were calculated as previously described (Nisley et al. 2025).

### Tissue and luminal content samples collection

On d 21 post-weaning, pigs utilized for nitrogen metabolism analysis were euthanized via captive bolt, followed by exsanguination. Thereafter, sections of ileum tissue (∼36 cm proximal from the ileo-cecal junction) and colon tissue (middle of ascending colon) were excised and fixed in 10% neutral buffered formalin for 24 h and then transferred to 70% ethanol and stored until further analysis. Intestinal contents from the colon were collected relative to the same region where their respective tissue were collected from and frozen at −80°C for later analysis.

### Intestinal morphology assessment

Sections of ileum and colon, stored in 70% ethanol, were trimmed and paraffin- embedded into blocks at the Iowa State University Veterinary Diagnostic Laboratory (Ames, IA). Ileum and colon were sectioned and stained with hematoxylin and eosin for intestinal morphology assessment as previously described by (Helm et al. 2020). Briefly, section intestinal slides were imaged at 20× magnification using an OLYMPUS BX 53/43 microscope equipped with a DP80 Olympus Camera (Olympic Scientific, Waltham, MA). For ileum tissue sections, villus height and crypt depth (μm) were determined from 15 to 20 randomly selected, well-oriented villus–crypt pairs using OLYMPUS CellSens Dimension 1.16 software (Olympus Scientific, Waltham, MA). The villus-to-crypt ratio was subsequently calculated from these measurements. Colonic morphology was evaluated by measuring the depth of 15–20 crypts per section following the same method.

### Volatile fatty acid and amine analysis

Colonic contents were thawed and 1.0 g of digesta from each sample was transferred to a 30 mL Nalgene polypropylene centrifuge tube and diluted with 5 mL of mQ water. Samples were then set horizontally in a Eberbach shaker 6010 (Eberbach Corporation, Van Buren Township, MI) and shaken at 280 osc/min overnight. Samples were then centrifuged at 21,000 × g for 23 min at 4°C. The supernatant was removed and placed into new tubes and o-phosphoric acid was added to achieve a pH of 2.5 to optimize VFA protonation for gas chromatography separation. Next, 1 mL of the pH-adjusted supernatant was placed into gas chromatography vials with 0.3 g of NaCl for gas chromatography analysis (Agilent 7890A GC, Agilent Technologies, Inc, Wilmington, DE) as described by Weber et al. (2010). Volatile fatty acid concentration is presented as micromole of VFA per gram of digesta.

For amine evaluation, 1.0 g of colon digesta was submitted to the W.M. Keck Metabolomics Research Laboratory (Iowa State University, Ames, IA) for targeted analysis of amines. Free amines were analyzed according to recommended methods of the EZ: faast Kit (Phenomenex, Torrance, CA) for free (physiological) AA analysis by GC-MS (Agilent 7890A GC, Agilent Technologies, Inc, Wilmington, DE) with minor modifications. Briefly, approximately 100 mg of colon contents were weighed into 1.5 mL microcentrifuge tubes and spiked with 100 μL of internal standard of reagent 1 (0.2 mM norvaline, 10% 1-propoanol, 20 mM hydrochloric acid) followed by the addition of 900 μL of ice cold 75% 1-popanol (HPLC grade 1-propanol 3:1 with LC-MS grade water, Fisher Scientific, Waltham, MA). Samples were homogenized with two 2.4 metals using a Bead Mill 24 Homogenizer (Fisher Scientific, Inc., Waltham, MA). Homogenized samples were then placed in an ice-cold sonicating water bath (Model 2510, Branson Ultrasonics, Brookfield, CT) for 10 min then centrifuged at 16,000 × g for 10 min. The supernatants were then recovered as sample extracts. Three hundred μL of each sample extract was subjected to solid phase extraction, derivatization, and GC-MS sample preparation according to the recommended methods of the EZ: faast Kit. Standards for putrescine and cadaverine (Sigma-Aldrich Co., St. Louis, MO) were prepared in the same manner as the samples. GC-MS analyses were performed with an Agilent 7890 gas chromatograph coupled to a model 5975 Mass Selective Detector (Agilent Technologies, Santa Clara, CA). A Zebron ZB- AAA column (10 m × 250 μm) was used (Phenomenex, Torrance, CA). One μL of sample was injected with the inlet operating in splitless mode and was held at a constant temperature of 250°C. The oven temperature was programmed as follows: an initial temperature of 70°C was increased to 320°C at a rate of 30°C/min and held for 1.667 min. Helium was used as a carrier gas at a flow rate of 1.2 mL/min. The MS transfer line was held at 310°C. Mass spectrometry detection was performed using electron ionization at 70 eV and source temperature and quadrupole temperature were set at 230°C and 150°C, respectively. The mass data were collected in the range from m/z 45 to m/z 450 across the entire run for all compounds, allowing for spectral fingerprint of each compound to be acquired. Identification and quantification were conducted using Automated Mass spectral Deconvolution and Identification System (AMDIS, National Institute of Standards and Technology, Gaithersburg, MD) with a manually curated retention indexed GC- MS library with additional identification performed using the NIST20 and Wiley 11 GC-MS spectral libraries (Phenomenex, Torrance, CA). Final quantification was calculated by integrating the corresponding peak area relative to the area of the internal standard and to the amine standards for each peak. Peaks without standards were quantified relative to internal standard only. Final quantified amine amounts were normalized to the colon content sample mass used in the extraction. Amines were measured on digesta samples from one replicate (*n* = 6/treatment).

### Statistical analysis

Data were analyzed using the GLIMMIX procedure of SAS v. 9.4 (SAS Institute, Cary, NC). All data were analyzed as a two-way ANOVA with the main effects of diet (HCP1.4, LCP1.4, and LCP1.2), NSPase (with or without xylanase), and their interaction with replicate included as a random effect. The pen was designated as the experimental unit for examining pig performance, fecal score, and mortality/removal data, while the individual pig served as the experimental unit for analyzing nitrogen retention, VFA, amine, and intestinal morphology data. These data were presented as least square means adjusted using Tukey’s HSD test to accommodate multiple comparisons. Categorical fecal consistency score data were analyzed using logistic regression with an ordinal distribution (0, 1, 2, and 3), and *P*-values were computed through residual pseudo-likelihood estimation and dual quasi-Newton optimization. Results are presented as the percentage of pens (%) that received scores of either “2” or “3” over the respective period, indicating diarrhea-like characteristics for clarity in interpretation. For all analyses, differences were considered significant when *p* < 0.05 and a tendency when 0.05* ≤ p* ≤ 0.10.

## Results

### Fecal scoring and general health

All pigs were PRRSV, PCV2, and *Mycoplasma pneumonia* vaccinated prior to arrival. Both groups of pigs were positive for hemolytic *Escherichia coli*. The aggregate of mortalities and removals for the population was 3.2% and did not differ between diet, NSPase, or their interaction (*P* > 0.10).

Overall, pigs fed the HCP1.4 diet had a higher proportion of diarrhea-like stools compared to LCP1.4 and LCP1.2-fed pigs (*P* < 0.001, Table 4). This occurrence was observed in all phases (all *p* < 0.05, respectively). During phase 1, a tendency for diet × NSPase interaction was observed as NSPase inclusion decreased diarrhea incidence in HCP1.4 pigs but increased diarrhea incidence in LCP1.4 and LCP1.2-fed pigs (*P* = 0.063).

**Table 4:** Effect of diet and NSPase inclusion on fecal score consistency.

		**LCP 1.2**		**LCP 1.4**		**HCP 1.4**		*P*-value^a^		
Parameter^b^	NSPase	No	Yes	No	Yes	No	Yes	Diet	NSPase	D × NSPase
***Diarrhea incidence^c^, %***										
** Phase 1, d 0–10**		53^y^	61^xy^	58^xy^	60^xy^	72^x^	65^xy^	0.045	0.453	0.063
** Phase 2, d 10–21**		1	2	2	2	7	8	<0.0001	0.891	0.304
** Phase 3, d 21–42**		2	0	1	2	11	8	<0.0001	0.637	0.706
**Overall, d 0–42**		21	25	23	23	32	29	<0.0001	0.917	0.289

a Diarrhea incidence rates were analyzed using a generalized linear model with an ordinal distribution.

b Estimates represented by 12 observations per treatment with pen as the experimental unit.

c Diarrhea incidence rate estimates are presented as the relative frequency of pens (%) that received scores of either “2” or “3,” which indicated diarrhea-like characteristics.

x, y Means lacking a common superscript tended to be different (*P* < 0.10).

### Growth performance

Start BW did not statistically differ among dietary treatments (*P* > 0.10, Table 5). On d 10, there was a tendency for an effect of diet on BW (*P* = 0.063), with pigs fed the HCP1.4 diet numerically 4% to 5% heavier than LCP1.4 and LCP1.2-fed pigs. Further, on d 21 and 42, diet affected BW (*P* < 0.001), with pigs fed HCP1.4 exhibiting numerically greater BW (9% to 10%) relative to pigs fed LCP diets. Overall (d 0–42), there was a tendency for a diet × NSPase interaction for feed efficiency (*P* = 0.066), where NSPase inclusion increased feed efficiency in LCP1.4-fed pigs but not in HCP1.4 and LCP1.2-fed pigs. No other diet × NSPase interactions were observed overall or by phase. Overall, diet affected both ADG and feed efficiency (both *p* < 0.001), with numerically greater growth and feed efficiency observed in HCP1.4-fed pigs. Further, there was a tendency for an effect of diet on ADFI (*P* = 0.066), with numerically greater intake in HCP1.4-fed pigs (0.62 vs. 0.59 kg).

**Table 5:** Effect of diet and NSPase inclusion on nursery pig performance.

		**LCP 1.2**		**LCP 1.4**		**HCP 1.4**			*P*-value		
Parameter^a^	NSPase	No	Yes	No	Yes	No	Yes	SEM	Diet	NSPase	D × NSPase
***Body weights, kg***											
**d 0**		6.2	6.4	6.3	6.2	6.4	6.2	0.19	0.820	0.690	0.285
**d 10**		8.4	8.7	8.6	8.6	9.1	8.9	0.34	0.063	0.789	0.373
**d 21**		12.0	12.8	12.8	12.7	13.8	13.6	0.32	<0.001	0.606	0.175
**d 42**		22.5	23.6	23.4	23.6	26.0	25.7	0.89	<0.001	0.358	0.321
***Phase 1, d 0–10***											
**ADG, kg**		0.21	0.23	0.23	0.24	0.27	0.27	0.017	<0.001	0.375	0.550
**ADFI, kg**		0.31	0.34	0.31	0.32	0.32	0.33	0.054	0.724	0.209	0.721
**G:F**		0.71	0.72	0.76	0.77	0.86	0.85	0.083	<0.001	0.918	0.854
***Phase 2, d 10–21***											
**ADG, kg**		0.34	0.35	0.37	0.37	0.42	0.42	0.023	<0.001	0.903	0.973
**ADFI, kg**		0.51	0.53	0.53	0.49	0.56	0.56	0.014	0.001	0.692	0.102
**G:F**		0.67	0.66	0.69	0.75	0.75	0.74	0.033	0.002	0.478	0.219
***Phase 3, d 21–42***											
**ADG, kg**		0.50	0.50	0.48	0.52	0.57	0.57	0.048	<0.001	0.363	0.499
**ADFI, kg**		0.88	0.96	0.95	0.95	0.95	0.99	0.060	0.083	0.035	0.365
**G:F**		0.56	0.53	0.51	0.54	0.60	0.58	0.023	0.002	0.497	0.198
***Overall, d 0–42***											
**ADG, kg**		0.35	0.36	0.36	0.37	0.42	0.42	0.019	<0.0001	0.285	0.542
**ADFI, kg**		0.57	0.61	0.59	0.59	0.61	0.63	0.014	0.058	0.134	0.247
**G:F**		0.62^z^	0.60^z^	0.61^z^	0.64^yz^	0.69^x^	0.67^xy^	0.026	<0.0001	0.650	0.066

a Estimates represented by 12 observations per treatment with pen as the experimental unit.

x,y,z Means lacking a common superscript tended to be different (*P* < 0.10).

During phase 1, diet affected ADG and feed efficiency (both *P* < 0.001), with numerically greater values observed in HCP1.4-fed pigs. During phase 2, diet affected ADG, ADFI, and feed efficiency (all *P* < 0.01), with numerically greater values observed in HCP1.4-fed pigs. In phase 3, diet affected ADG (*P* < 0.001) and feed efficiency (*P* = 0.001), and tended to affect ADFI (*P* = 0.084). Additionally, in phase 3, NSPase inclusion increased ADFI (*P* = 0.039).

### Nitrogen digestibility and retention

Nitrogen intake (g/d) was affected by diet (*P* < 0.001; Table 6), with greater intake observed in HCP1.4-fed pigs, driven by the inherently higher nitrogen concentration of the diet. Furthermore, diet affected ATTD of nitrogen (*P* < 0.001), with numerically greater coefficients observed in HCP1.4-fed pigs. Fecal nitrogen excretion (g/d) did not differ among diets (*P* > 0.10), whereas diet affected urinary nitrogen excretion (*P* < 0.001), with numerically greater values observed in HCP1.4-fed pigs. Similarly, diet affected total nitrogen excretion (*P* < 0.001), with numerically greater excretion observed in HCP1.4-fed pigs. There was a tendency for a diet × NSPase interaction for nitrogen retention (g/d; *P* = 0.053), where NSPase inclusion decreased retention in LCP1.4-fed pigs but increased retention in LCP1.2 and HCP1.4-fed pigs. Overall, diet affected nitrogen retention (*P* < 0.001), with numerically greater retention observed in HCP1.4-fed pigs. Lastly, diet affected nitrogen retained as a percent of intake (*P* = 0.015), with numerically lower values observed in LCP1.2-fed pigs.

**Table 6:** Effect of diet and NSPase inclusion on nitrogen digestibility and retention.

		LCP1.2		LCP1.4		HCP1.4			*P*-value		
Parameter^a^	NSPase	No	Yes	No	Yes	No	Yes	SEM	Diet	NSPase	D × NSPase
***Digestibility***											
**Gross energy, %**		87.7	87.6	88.0	87.3	89.7	89.5	0.84	0.005	0.522	0.874
***Nitrogen metabolism***											
**Nitrogen intake, g/d**		11.5^c^	11.4^c^	12.8^c^	12.0^c^	15.2^b^	15.3^b^	0.42	<0.001	0.087	0.010
**Nitrogen ATTD, %**		83.9	83.5	85.1	82.9	87.7	88.1	1.00	<0.001	0.290	0.280
***Nitrogen excretion***											
**Fecal, g/d**		1.84	1.88	1.91	2.05	1.87	1.84	0.189	0.482	0.594	0.770
**Urine, g/d**		2.33	1.90	1.29	1.45	3.12	2.67	0.326	<0.001	0.380	0.568
**Total, g/d**		4.17	3.78	3.19	3.50	4.99	4.52	0.312	<0.001	0.479	0.401
***Nitrogen retention***											
**N retention, g/d**		7.28^z^	7.63^z^	9.57^xy^	8.48^yz^	10.20^z^	10.83^x^	0.441	<0.001	0.922	0.053
**N retained, % of intake**		63.4	66.9	74.7	70.8	67.1	70.5	2.46	0.015	0.639	0.242

a Estimates represented by 12 observations per treatment with pig as the experimental unit, d 17–21 of study.

b,c Means lacking a common superscript are significantly different (*P* < 0.05).

x,y,z Means lacking a common superscript tended to be different (*P* < 0.10).

### Ileal and colonic morphology

Ileum villus height did not differ between diet, NSPase inclusion, or their interaction (*P* > 0.10; Table 7). There was a tendency for a NSPase inclusion to increase ileal crypt depth exclusively in HCP1.4-fed pigs (*P* = 0.085). No other main effects or diet × NSPase interactions were observed among villus/crypt ratio or colon crypt depth measurements.

**Table 7:** Effect of diet and NSPase inclusion on intestinal morphology at d 21.

		**LCP 1.2**		**LCP 1.4**		**HCP 1.4**			*P*-value		
Parameter^a^	NSPase	No	Yes	No	Yes	No	Yes	SEM	Diet	NSPase	D × NSPase
***Ileum***											
**Villus height, µm**		394	376	403	392	351	368	19.0	0.134	0.813	0.645
**Crypt depth, µm**		251^xy^	245^xy^	275^x^	240^xy^	235^y^	274^x^	17.8	0.829	0.935	0.085
**Villus: crypt ratio**		1.68	1.57	1.51	1.75	1.53	1.42	0.128	0.376	0.945	0.281
***Colon***											
**Crypt depth, µm**		517	485	502	484	518	548	24.6	0.232	0.748	0.424

a Estimates represented by 12 observations per treatment with pig as the experimental unit.

x,y Means lacking a common superscript tended to be different (*P* < 0.10).

### Colonic volatile fatty acid and amine concentrations

Volatile fatty acids measured included SCFAs (acetic acid, propionic acid, butyric acid, valeric acid, and caproic acid), branched-SCFAs (isobutyric acid, isovaleric acid, and isocaproic acid), and the medium-chain fatty acid (MCFA) heptanoic acid. Overall, no diet × NSPase interactions or NSPase effect were observed (*P* > 0.10; Table 8). Acetic acid and propionic acid both tended to be affected by diet (*P* = 0.074 and *p* = 0.065, respectively), with numerically greater concentrations observed in HCP1.4-fed pigs. Valeric acid concentration was affected by diet (*P* = 0.045), with numerically lower concentrations observed in LCP1.2-fed pigs (∼45% lower). Branched-chain SCFAs isobutyric acid and isocaproic acid tended to be affected by diet (*P* = 0.053 and *p* = 0.060, respectively), with numerically greater concentrations observed in HCP1.4-fed pigs. Further, branched-chain SCFA isovaleric acid concentration was affected by diet (*P* = 0.044), with numerically lower concentrations observed in LCP1.2-fed pigs. Finally, diet affected total VFA concentration (*P* = 0.027), with numerically greater overall VFA production observed in HCP1.4-fed pigs (13 and 26% greater than LCP1.2 and LCP1.4-fed pigs, respectively).

**Table 8:** Effect of diet and NSPase inclusion on colonic luminal concentrations of VFA.

		LCP1.2		LCP1.4		HCP1.4			*P*-value		
Parameter^a^	NSPase	No	Yes	No	Yes	No	Yes	SEM	Diet	NSPase	D × NSPase
***Colonic digesta VFA, μmol/g***											
**Acetic acid**		119	111	105	136	152	166	26.8	0.074	0.467	0.653
**Propionic acid**		16.7	17.5	18.2	19.1	22.4	21.6	2.09	0.065	0.868	0.907
**Butyric acid**		12.0	12.8	16.8	15.0	18.1	11.4	3.94	0.312	0.182	0.270
**Isobutyric acid**		0.81	0.75	1.54	1.29	2.77	1.48	0.671	0.053	0.229	0.467
**Isovaleric acid**		0.98	0.96	2.27	2.32	3.18	2.06	0.777	0.044	0.517	0.631
**Valeric acid**		0.65	0.90	1.29	1.49	1.52	1.32	0.377	0.045	0.719	0.703
**Isocaproic acid**		0.18(6/12)	0.21(8/12)	0.95(6/12)	0.76(6/12)	2.44(7/12)	0.81(7/12)	1.042	0.060	0.227	0.306
**Caproic acid**		0.17	0.19	0.25	0.27	0.74	0.28	0.159	0.113	0.293	0.229
**Heptanoic acid**		0.04(10/12)	0.04(10/12)	0.18(10/12)	0.14	0.13	0.10(11/12)	0.084	0.106	0.643	0.938
**Total VFA**		140	145	147	176	202	205	32.7	0.027	0.513	0.814

a Estimates represented by 12 observations per treatment with pig as the experimental unit. For variables with concentrations below the limit of detection, the number of pigs with detectable values is indicated in parentheses (*n*/12) next to the marginal means. Samples below the detection limit were assigned a value of 0 prior to analysis.

Day 22 colonic digesta analysis of polyamines cadaverine and putrescine concentrations observed no diet × NSPase interactions (*P* > 0.10, Table 9). While NSPase inclusion tended to decrease colonic cadaverine concentration by ∼49% (*P* = 0.079), no other NSPase effects were observed on individual amine concentration (*P* > 0.10). Essential amino acids (EAAs; Lys, Leu, Val, and Ile) exhibited diet × NSPase interactions, with concentrations increasing in HCP1.4-fed pigs but decreasing in LCP1.2 and LCP1.4-fed pigs with NSPase (all *p* < 0.05), and a similar trend observed for Trp (*P* = 0.051). Colonic digesta concentrations of Val, Leu, Ile, and Trp differed among diets (all *p* < 0.05), with LCP1.4-fed pigs having the highest concentrations. Amine and non-essential amino acid (NEAA) concentrations, including Ala, Gly, and Orn, were affected by diet (all *P* < 0.05), with numerically lower concentrations observed in LCP1.2-fed pigs. Tyrosine tended to be affected by diet (*P* = 0.059), with numerically greater concentrations observed in LCP1.4-fed pigs. Among these NEAAs, NSPase inclusion increased Gly concentration and tended to increase Orn concentration only in HCP1.4-fed pigs (*P* = 0.009 and *p* = 0.069, respectively). Alpha amine aspartic acid concentrations increased in HCP1.4 and LCP1.2-fed pigs with NSPase inclusion whereas in LCP1.4-fed pigs they decreased (*P* = 0.025). Additionally, Asp tended to be lowest in LCP1.2-fed pigs (*P* = 0.056). Lastly, all measured amines were summated to provide total amine concentration. No diet by NSPase or diet effects were observed, although, NSPase inclusion tended to reduce overall amine concentrations by 20% to 60%, primarily driven by NSPase inclusions impact on cadaverine concentration (*P* = 0.077).

**Table 9:** Effect of diet and NSPase inclusion on colonic luminal concentrations of amines.

		LCP1.2		LCP1.4		HCP1.4			*P*-value		
Parameter^a^	NSPase	No	Yes	No	Yes	No	Yes	SEM	Diet	NSPase	D × NSPase
***Colonic digesta amine, nmol/mg***											
**Cadaverine**		5.36	3.85	9.08	4.43	6.32	2.21	2.239	0.501	0.079	0.774
**Putrescine**		2.02	1.82	1.98	1.14	1.49	1.86	0.493	0.768	0.598	0.474
**Lys**		0.68^bc^	0.65^c^	0.89^b^	0.71^bc^	0.67^bc^	0.86^b^	0.072	0.182	0.912	0.049
**Met**		0.14	0.16	0.16	0.11	0.10	0.12	0.021	0.305	0.943	0.117
**Leu**		0.30^c^	0.28^c^	0.51^b^	0.35^bc^	0.33^bc^	0.40^bc^	0.044	0.012	0.366	0.041
**Ile**		0.20^a^	0.21^a^	0.37^b^	0.23^c^	0.22^c^	0.29^bc^	0.032	0.029	0.450	0.007
**Thr**		0.32^y^	0.35^xy^	0.46^x^	0.32^y^	0.33^y^	0.42^xy^	0.053	0.566	0.887	0.094
**Val**		0.30^c^	0.28^c^	0.46^b^	0.33^bc^	0.32^bc^	0.40^bc^	0.038	0.027	0.385	0.029
**His**		0.01	0.01	0.02	0.03	0.01	0.01	0.004	0.009	0.285	0.648
**Phe**		0.17	0.17	0.26	0.19	0.18	0.21	0.022	0.057	0.422	0.152
**Trp**		0.014^y^	0.017^xy^	0.035^x^	0.020^xy^	0.014^y^	0.024^xy^	0.0050	0.047	0.789	0.051
**Ala**		0.98	0.91	1.17	1.14	1.18	1.29	0.114	0.048	0.982	0.710
**Gln**		0.10	0.08	0.17	0.14	0.13	0.17	0.032	0.102	0.989	0.571
**Pro**		0.29	0.26	0.41	0.28	0.33	0.33	0.046	0.348	0.163	0.348
**Gly**		0.40^c^	0.40^c^	0.56^b^	0.40^c^	0.44^bc^	0.55^b^	0.039	0.043	0.584	0.009
**Tyr**		0.17	0.17	0.25	0.20	0.18	0.19	0.023	0.059	0.487	0.285
**Orn**		0.13^y^	0.10^y^	0.16^xy^	0.12^y^	0.19^xy^	0.28^x^	0.03	0.001	0.725	0.069
**Asp**		0.35^c^	0.45^bc^	0.85^b^	0.39^c^	0.61^bc^	0.81^b^	0.122	0.056	0.602	0.025
**Glu**		1.5	1.4	2.0	1.4	1.8	2.0	0.20	0.085	0.521	0.176
**Pipecolic acid**		0.43	0.23	0.21	0.48	0.40	0.14	0.129	0.809	0.542	0.104
**Total amines**		13.8	11.8	20.1	12.5	15.3	12.6	2.66	0.442	0.077	0.533

a Estimates represented by 6 observations per treatment with pig as the experimental unit.

b,c Means lacking a common superscript are significantly different (*P* < 0.05).

x, y Means lacking a common superscript tended to be different (*P* < 0.10).

## Discussion

Enteric disturbances over the early nursery period have been shown to be mitigated by formulating LCP diets (Luise et al. 2021). Typically, higher inclusion of crystalline amino acids and specialty proteins or reduced SID AA curves are utilized by nutritionists to reduce CP content in early nursery diets. The increased incidence of PWD in pigs fed HCP diets has been linked to cytotoxic byproducts of proteolytic fermentation in the hindgut (Zhang et al. 2020). Increasing fiber fermentability through NSPase supplementation may mitigate this by favoring saccharolytic over proteolytic fermentation. Therefore, herein the objective was to evaluate how industry relevant HCP and LCP diets, with or without a NSPase (xylanase), would impact nursery pig performance, nitrogen balance, hindgut fermentation and markers of intestinal health. To test this, three diets were formulated: a HCP (24% CP) diet with a 1.4% Lys curve, a LCP (17% CP) with a 1.4% SID Lys curve, and a LCP (16% CP) with a lower Lys curve (1.2% SID Lys).

The occurrence of diet induced PWD has been extensively documented and studied in relation to dietary CP content (Wang et al. 2018; Luise et al. 2021). Herein, pigs fed HCP diets observed higher incidences of diarrhea compared to pigs fed either of the LCP diets. These findings align with observations reported in previous studies (Limbach et al. 2021; Lynegaard et al. 2021; Kim et al. 2023). Interestingly, NSPase supplementation reduced diarrhea incidence in HCP1.4-fed pigs during phase 1 (d 0–10), but increased diarrhea incidence in LCP1.2 or LCP1.4 pigs. This effect may suggest that NSPase supplementation increased release of carbohydrate-bound proteins in soybean meal, as HCP1.4 diets contained 37.45% soybean meal versus 10% to 11.85% in LCP diets, thereby lowering proteolytic fermentation and PWD. This aligns with Acosta et al. (2025), who reported that weanling pigs fed diets containing 20% soybean meal had reduced phase 1 (d 1–14) diarrhea incidence when supplemented with xylanase, accompanied by a marginal improvement in ATTD of CP. However, the authors cannot reconcile the observed increase in diarrhea incidence with NSPase supplementation in LCP-fed pigs and are unaware of any literature supporting this response. Due to the latter described HCP mediated increase in PWD, HCP diets have been suspected to potentially antagonize pig livability. In this study, pigs fed a HCP diet had no reportable deleterious effects on pig livability compared to pigs fed LCP diets.

Although increased PWD incidence was observed in pigs fed a HCP diet, the HCP1.4-fed pigs had the highest ADG and feed efficiency compared to LCP1.4 and LCP1.2-fed pigs through all phases and overall. These results corroborate prior research that reported a reduction in ADG in weaned pigs fed diets containing 16% to 17% CP compared to their respective HCP diet counterparts (Nyachoti et al. 2006; Cho et al. 2008). Despite the presence of different SID Lys curves, surprisingly, no differences in growth performance were observed between pigs fed the LCP1.4 and LCP1.2 diets. The observed attenuated growth performance in pigs fed LCP diets can be reasonably attributed to marginal deficiencies in amino acids, which are vital for facilitating optimal lean growth. Furthermore, SID Lys: CP ratios above 6.50 have been shown to increase the risk of inadvertent NEAA and EAA deficiencies (Millet et al. 2018), and both LCP diets exceeded 6.50 SID Lys: CP (≥6.85 and ≥ 7.71 SID Lys: CP for LCP1.2 and LCP1.4, respectively). This trend has been continually documented in nursery pig studies that evaluate LCP diets (Nyachoti et al. 2006; Spring et al. 2020), where reducing CP below 18.4% often results in impaired growth performance (Rocha et al. 2022). Herein, growth performance parameters were not altered when NSPase was included in the diets. While xylanase supplementation in pig diets has the capacity to improve nutrient digestibility and feed efficiency, it does not consistently translate into a measurable increase in pig performance (Duarte et al. 2019; Torres-Pitarch et al. 2019).

Pigs fed the HCP1.4 diet had the highest nitrogen ATTD coefficient, aligning with previous research, which reported that pigs fed HCP diets generally display higher nitrogen ATTD compared to pigs fed lower CP diets when the diets are corn-soybean meal based (Kephart and Sherritt 1990; Kerr and Easter 1995). Furthermore, pigs fed the HCP1.4 diets had the highest nitrogen excretion, corroborating previous findings (Kirchgessner and Roth 1993; Portejoie et al. 2004; Shurson and Kerr 2023). The pigs fed LCP1.4 diets demonstrated the highest nitrogen retention coefficient, whereas the pigs fed LCP1.2 diets exhibited the lowest nitrogen retention coefficient. This discrepancy could likely be attributed to an amino acid deficiency that hindered the optimal accretion of lean tissue in LCP1.2-fed pigs, often observed in Lys restricted pigs (Hasan et al. 2020). Furthermore, there was no discernible impact of the diets on gut morphology parameters, and these parameters appeared to have no correlation with nutrient retention, feed efficiency, or nitrogen digestibility in the work presented herein. The insignificance in gut morphology aligns with previous work that investigated the effects of different CP levels in weaned pig diets (Opapeju et al. 2008; Luise et al. 2021). Moreover, previous work has shown negligible changes in colonic epithelial barrier function between nursery pigs fed either a high (24%) or low (17%) CP diet (Pearce et al. 2024).

Proteolytic fermentation, defined as the microbial degradation of proteins and peptides in the cecum and colon, produces metabolites including SCFA, biogenic amines, ammonia, p-cresols, phenols, indoles, and hydrogen sulfide. Among the three major SCFAs, acetic acid plays a crucial role in lipid biosynthesis (Macfarlane and Macfarlane 2012), propionic acid can be utilized in gluconeogenesis (Adler et al. 2021), and butyric acid serves as an important energy source for colonocytes (Holscher 2017; Gardiner et al. 2020), promoting cell proliferation and maintaining tight junction integrity (Lupton 2004; Peng et al. 2009). Butyrate is often regarded as the most beneficial SCFA, yet its production appears less responsive to CP intake compared with acetate and propionate (Walker et al. 2005). Consistent with this, the current study found that increased hindgut protein supply elevated acetate and propionate but did not enhance butyrate production. Furthermore, SCFAs can lower the pH of the colonic lumen which can inhibit the growth of pathogenic microorganisms (Macfarlane and Macfarlane 2012; Zhang et al. 2020). In this study, pigs receiving the HCP1.4 diet had the greatest total colonic SCFA concentrations compared with those on either LCP diet, suggesting a potential benefit of soybean meal-based HCP diets in nursery pigs. Non-starch polysaccharidase supplementation did not alter VFA production, likely due to the predominance of insoluble xylan-bound xylose within NSP in corn (2 g/kg soluble; 28 g/kg insoluble) and soybean meal (2 g/kg soluble; 17 g/kg insoluble; Knudsen 1997). While NSPase may facilitate partial hydrolysis of these NSP, improving nutrient accessibility, this likely did not generate sufficient free sugars to alter VFA production.

Polyamines such as putrescine support colonic mucosal growth, intestinal barrier integrity, and repair of mucosal injury (Löser et al. 1999; Tan et al. 2024). However, putrescine has also been reported to suppress MCT-1 transporter expression, increase expression of pro-inflammatory cytokines IL-8 and TNF-α, and, along with cadaverine, exhibit cytotoxic effects in vitro (Villodre Tudela et al. 2015; del Rio et al. 2019). Cadaverine and putrescine concentrations did not differ among CP diets, indicating that modern dietary CP levels may exert less influence on colonic polyamine production than previously reported, where dietary CP explained variation in cadaverine (*R*^2^ = 34.7, *P* < 0.05) and putrescine (*R*^2^ = 23.3, *P* < 0.05) concentrations (Luise et al. 2021). However, NSPase supplementation reduced colonic cadaverine production, suggesting NSPase supplementation may help mitigate hindgut proteolytic fermentation. This finding may be attributed to xylanases capacity to reduce luminal viscosity and liberate fiber-bound proteins (Vasanthakumari et al. 2023), particularly considering the soybean meal content in these diets (HCP1.4, 38%; LCP1.4, 15%; and LCP1.2, 18%). The inconsistencies observed between dietary CP-driven proteolytic fermentation and its metabolic byproducts in relation to pig health emphasize the complexity of these interactions and the need for continued investigation, as noted by Pieper et al. (2016).

In this study, the first null hypothesis was partially rejected as HCP-fed pigs supplemented with NSPase observed altered colonic fermentation and reduced PWD incidence without improvement to feed efficiency. Additionally, the second null hypothesis was rejected as HCP-fed pigs had increased PWD incidence, nitrogen excretion, and SCFA production, without attenuating growth. Finally, the third null hypothesis could not be rejected as both LCP diets observed similar growth performance and PWD incidence. In conclusion, modern perspectives on weanling-pig nutrition suggest lowering dietary CP as a paradigm to reduce PWD and nitrogen excretion. However, reducing CP while attempting to standardize SID Lys and SID AA: Lys ratios can manifest unexpected NEAA and EAA deficiencies and attenuate pig growth. Moreover, although an increase in protein fermentation metabolites may be observed, increasing proteolytic fermentation to the extent that is observed in swine fed practical diets may have no physiologically negative impact to the pig. Nevertheless, as CP intake directly correlates to nitrogen excretion, production systems must account for increased nitrogen output. Finally, realizing growth performance or fermentation benefits from NSPase supplementation may require a sufficient level of enzyme-accessible NSP.

## List of abbreviations

AA: amino acid
ADG: average daily gain
ADFI: average daily feed intake
ATTD: apparent total tract digestibility
BW: bodyweight
CEAA: conditionally essential amino acid
CP: crude protein
EAA: essential amino acid
GE: gross energy
G:F: gain: feed
HCP: high crude protein
LCP: low crude protein
MCFA: medium-chain fatty acid
NEAA: non-essential amino acid
NSP: non-starch polysaccharide
NSPase: non-starch polysaccharidase
PWD: post-weaning diarrhea
SCFA: short-chain fatty acid
SID: standardized ileal digestibility
VFA: volatile fatty acid

## References

[txag090-B1] Acosta J. P. , Espinosa C. D., González-Ortiz G., Stein H. H. 2025. Growth performance and total tract digestibility of nutrients for weanling pigs are improved by an exogenous xylanase and a stimbiotic regardless of maternal xylanase consumption. J. Anim. Sci. Biotechnol. 16:68. 10.1186/s40104-025-01205-w 40375102 PMC12080015

[txag090-B2] Adeola O. 2001. Digestion and balance techniques in pigs. In: Lewis A. J. Southern L. L., editors. Swine nutrition. 2^nd^ ed. CRC Press, Washington, DC. p. 903–916.

[txag090-B3] Adler G. K. et al 2021. Acute effects of the food preservative propionic acid on glucose metabolism in humans. BMJ Open Diabetes Res. Care. 9:e002336. 10.1136/bmjdrc-2021-002336 PMC8314753 34312159

[txag090-B4] Chen Y . et al 2020. Supplementation of non-starch polysaccharide enzymes cocktail in a corn-miscellaneous meal diet improves nutrient digestibility and reduces carbon dioxide emissions in finishing pigs. Animals. 10:232. 10.3390/ani10020232 32024084 PMC7070354

[txag090-B5] Cho J. H. et al 2008. Effects of reducing dietary crude protein on growth performance, odor gas emission from manure and blood urea nitrogen and IGF-1 concentrations of serum in nursery pigs. Anim. Sci. J. 79:453–459. 10.1111/j.1740-0929.2008.00549.x

[txag090-B6] del Rio B . et al 2019. The biogenic amines putrescine and cadaverine show in vitro cytotoxicity at concentrations that can be found in foods. Sci. Rep. 9:120. 10.1038/s41598-018-36239-w 30644398 PMC6333923

[txag090-B7] Duarte M. E. , Zhou F. X., Dutra W. M., Kim S. W. 2019. Dietary supplementation of xylanase and protease on growth performance, digesta viscosity, nutrient digestibility, immune and oxidative stress status, and gut health of newly weaned pigs. Anim. Nutr. 5:351–358. 10.1016/j.aninu.2019.04.005 31890911 PMC6920460

[txag090-B8] Gardiner G. E. , Metzler-Zebeli B. U., Lawlor P. G. 2020. Impact of intestinal microbiota on growth and feed efficiency in pigs: a review. Microorganisms. 8:1886. 10.3390/microorganisms8121886 33260665 PMC7761281

[txag090-B9] Hasan M. S. , Crenshaw M. A., Liao S. F. 2020. Dietary lysine affects amino acid metabolism and growth performance, which may not involve the GH/IGF-1 axis, in young growing pigs. J. Anim. Sci. 98:skaa004. 10.1093/jas/skaa004 31922564 PMC6986777

[txag090-B10] Helm E. T. et al 2020. Impact of viral disease hypophagia on pig jejunal function and integrity. PLoS One. 15:e0227265. 10.1371/journal.pone.0227265 31910236 PMC6946155

[txag090-B11] Holscher H. D. 2017. Dietary fiber and prebiotics and the gastrointestinal microbiota. Gut Microbes. 8:172–184. 10.1080/19490976.2017.1290756 28165863 PMC5390821

[txag090-B12] Kephart K. B. , Sherritt G. W. 1990. Performance and nutrient balance in growing swine fed low-protein diets supplemented with amino acids and potassium. J. Anim. Sci. 68:1999–2008. 10.2527/1990.6871999x 2384390

[txag090-B13] Kerr B. J. , Easter R. A. 1995. Effect of feeding reduced protein, amino acid-supplemented diets on nitrogen and energy balance in grower pigs. J. Anim. Sci. 73:3000–3008. 10.2527/1995.73103000x 8617671

[txag090-B14] Kim H. , Shin H., Kim Y. Y. 2023. Effects of different levels of dietary crude protein on growth performance, blood profiles, diarrhea incidence, nutrient digestibility, and odor emission in weaning pigs. Anim. Biosci. 36:1228–1240. 10.5713/ab.22.0440 36915927 PMC10330979

[txag090-B15] Kirchgessner M , Roth F. X. 1993. Lowering of the nitrogen excretion in swine. Arch Tierernahr. 43:283–301. 10.1080/17450399309386044 8517772

[txag090-B16] Knudsen K. E. B. 1997. Carbohydrate and lignin contents of plant materials used in animal feeding. Anim. Feed Sci. Technol. 67:319–338. 10.1016/S0377-8401(97)00009-6

[txag090-B17] Limbach J. R. , Espinosa C. D., Perez-Calvo E., Stein H. H. 2021. Effect of dietary crude protein level on growth performance, blood characteristics, and indicators of intestinal health in weanling pigs. J. Anim. Sci. 99:1–14. 10.1093/jas/skab166 PMC8202089 34019637

[txag090-B18] Liu S . et al 2019. β-xylosidase and β-mannosidase in combination improved growth performance and altered microbial profiles in weanling pigs fed a corn-soybean meal-based diet. Asian-Australas. J. Anim. Sci. 32:1734–1744. 10.5713/ajas.18.0873 31010999 PMC6817776

[txag090-B19] Löser C. , Eisel A., Harms D., Fölsch U. R. 1999. Dietary polyamines are essential luminal growth factors for small intestinal and colonic mucosal growth and development. Gut. 44:12–16. 10.1136/gut.44.1.12 9862820 PMC1760068

[txag090-B20] Luise D. , Chalvon-Demersay T., Lambert W., Bosi P., Trevisi P. 2021. Meta-analysis to evaluate the impact of the reduction of dietary crude protein on the gut health of post- weaning pigs. Ital. J. Anim. Sci. 20:1386–1397. 10.1080/1828051X.2021.1952911

[txag090-B21] Lupton J. R. 2004. Microbial degradation products influence Colon cancer risk: the butyrate controversy. J. Nutr. 134:479–482. 10.1093/jn/134.2.479 14747692

[txag090-B22] Lynegaard J. C. et al 2021. Low protein diets without medicinal zinc oxide for weaned pigs reduced diarrhea treatments and average daily gain. Animal. 15:100075. 10.1016/j.animal.2020.100075 33516025

[txag090-B23] Macfarlane G. T. , Gibson G. R., Cummings J. H. 1992. Comparison of fermentation reactions in different regions of the human colon. J. Appl. Bacteriol. 72:57–64. 10.1111/j.1365-2672.1992.tb04882.x 1541601

[txag090-B24] Macfarlane G. T. , Macfarlane S. 2012. Bacteria, colonic fermentation, and gastrointestinal health. J. AOAC. Int. 95:50–60. 10.5740/jaoacint.SGE_Macfarlane 22468341

[txag090-B25] Millet S . et al 2018. The effect of crude protein reduction on performance and nitrogen metabolism in piglets (four to nine weeks of age) fed two dietary lysine levels. J. Anim. Sci. 96:3824–3836. 10.1093/jas/sky254 29939350 PMC6127758

[txag090-B26] Nisley M. J. et al 2025. Increasing dietary soybean-derived trypsin inhibitor protein compromises nursery pig performance, nitrogen digestibility and retention. J. Anim. Sci. 103:skaf253. 10.1093/jas/skaf253 40808373 PMC12405685

[txag090-B27] National Research Council. 2012. Nutrient requirements of swine. 11^th^ rev. ed. Natl. Acad. Press, Washington, DC. 10.17226/13298

[txag090-B28] Nyachoti C. M. , Omogbenigun F. O., Rademacher M., Blank G. 2006. Performance responses and indicators of gastrointestinal health in early-weaned pigs fed low-protein amino acid-supplemented diets. J. Anim. Sci. 84:125–134. 10.2527/2006.841125x 16361499

[txag090-B30] Opapeju F. O. , Rademacher M., Blank G., Nyachoti C. M. 2008. Effect of low-protein amino acid-supplemented diets on the growth performance, gut morphology, organ weights and digesta characteristics of weaned pigs. Animal. 2:1457–1464. 10.1017/S175173110800270X 22443903

[txag090-B31] Peng L. , Li Z.-R., Green R. S., Holzman I. R., Lin J. 2009. Butyrate enhances the intestinal barrier by facilitating tight junction assembly via activation of amp-activated protein kinase in caco-2 cell monolayers. J. Nutr. 139:1619–1625. 10.3945/jn.109.104638 19625695 PMC2728689

[txag090-B32] Pearce S. C. , Nisley M. J., Kerr B. J., Sparks C., Gabler N. K. 2024. Effects of dietary protein level on intestinal function and inflammation in nursery pigs. J. Anim. Sci. 102:skae077. 10.1093/jas/skae077 38504643 PMC11015048

[txag090-B33] Petry A. L. , Patience J. F. 2020. Xylanase supplementation in corn-based swine diets: a review with emphasis on potential mechanisms of action. J. Anim. Sci. 98:skaa318. 10.1093/jas/skaa318 32970148 PMC7759750

[txag090-B34] Pieper R . et al 2016. Health relevance of intestinal protein fermentation in young pigs. Anim. Health Res. Rev. 17:137–147. 10.1017/S1466252316000141 27572670

[txag090-B35] Portejoie S. , Dourmad J. Y., Martinez J., Lebreton Y. 2004. Effect of lowering dietary crude protein on nitrogen excretion, manure composition and ammonia emission from fattening pigs. Livest. Prod. Sci. 91:45–55. 10.1016/j.livprodsci.2004.06.013

[txag090-B36] Rocha G. C. , Duarte M. E., Kim S. W. 2022. Advances, implications, and limitations of low-crude-protein diets in pig production. Animals. 12:3478. 10.3390/ani12243478 36552397 PMC9774321

[txag090-B37] Shurson G. C. , Kerr B. J. 2023. Challenges and opportunities for improving nitrogen utilization efficiency for more sustainable pork production. Front. Anim. Sci. 4:1204863. 10.3389/fanim.2023.1204863

[txag090-B38] Spring S . et al 2020. Effect of very low-protein diets supplemented with branched-chain amino acids on energy balance, plasma metabolomics and fecal microbiome of pigs. Sci. Rep. 10:15859. 10.1038/s41598-020-72816-8 32985541 PMC7523006

[txag090-B39] Tan B. , Xiao D., Wang J., Tan B. 2024. The roles of polyamines in intestinal development and function in piglets. Animals (Basel). 14:1228. 10.3390/ani14081228 38672376 PMC11047586

[txag090-B40] Torres-Pitarch A. , Manzanilla E. G., Gardiner G. E., O’Doherty J. V., Lawlor P. G. 2019. Systematic review and meta-analysis of the effect of feed enzymes on growth and nutrient digestibility in grow-finisher pigs: effect of enzyme type and cereal source. Anim. Feed. Sci. Technol. 251:153–165. 10.1016/j.anifeedsci.2018.12.007

[txag090-B41] Vasanthakumari B. L. et al 2023. A new monocomponent xylanase improves performance, ileal digestibility of energy and nutrients, intestinal morphology, and intestinal microbiota in young broilers. J. Appl. Poult. Res. 32:100301. 10.1016/j.japr.2022.100301

[txag090-B42] Villodre Tudela C . et al 2015. Down-regulation of monocarboxylate transporter 1 (MCT1) gene expression in the colon of piglets is linked to bacterial protein fermentation and pro- inflammatory cytokine-mediated signaling. Br. J. Nutr. 113:610–617. 10.1017/S0007114514004231 25656974

[txag090-B43] Walker A. W. , Duncan S. H., McWilliam Leitch E. C., Child M. W., Flint H. J. 2005. pH and peptide supply can radically alter bacterial populations and short-chain fatty acid ratios within microbial communities from the human colon. Appl. Environ. Microbiol. 71:3692–3700. 10.1128/AEM.71.7.3692-3700.2005 16000778 PMC1169066

[txag090-B44] Wang Y . et al 2018. Advances in low-protein diets for swine. J. Anim. Sci. Biotechnol. 9:60–14. 10.1186/s40104-018-0276-7 30034802 PMC6052556

[txag090-B45] Weber T. E. , Trabue S. L., Ziemer C. J., Kerr B. J. 2010. Evaluation of elevated dietary corn fiber from corn germ meal in growing female pigs. J. Anim. Sci. 88:192–201. 10.2527/jas.2009-1896 19783691

[txag090-B46] Zhang H . et al 2020. Impact of fermentable protein, by feeding high protein diets, on microbial composition, microbial catabolic activity, gut health and beyond in pigs. Microorganisms. 8:1735. 10.3390/microorganisms811173 33167470 PMC7694525

